# Association of the hemoglobin glycation index with the large-artery atherosclerosis subtype in ischemic stroke: a dual-cohort study

**DOI:** 10.3389/fneur.2026.1841295

**Published:** 2026-06-29

**Authors:** Xinyu Tong, Jianxiong Gu, Chuxin Lyu, Yichun Zhao, Lei Chen, Tianzhi Ren, Haoxin Wu, Lei Yu, Ying Rui

**Affiliations:** 1 Department of Neurology, Wuxi Affiliated Hospital of Nanjing University of Chinese Medicine, Wuxi, China; 2 College of Traditional Chinese Medicine, Nanjing University of Chinese Medicine, Nanjing, Jiangsu, China; 3 First Clinical Medical School, Nanjing University of Chinese Medicine, Nanjing, Jiangsu, China; 4 Department of Clinical Psychology, Jiangyin Third People's Hospital, Wuxi, Jiangsu, China

**Keywords:** hemoglobin glycation index, ischemic stroke, large-artery atherosclerosis, Mendelian randomization, MIMIC-IV

## Abstract

**Background:**

The hemoglobin glycation index (HGI) reflects discordance between measured HbA1c and glucose-predicted HbA1c, but in acute ischemic stroke (IS) it may also capture stress-hyperglycemia-related fasting plasma glucose (FPG) elevation. We examined the association of HGI with large-artery atherosclerosis (LAA) and in-hospital mortality.

**Methods:**

We retrospectively analyzed 4,500 IS patients from MIMIC-IV and 330 patients from an external clinical cohort. HGI was calculated using cohort-specific FPG-HbA1c regression equations. Multivariable logistic regression and restricted cubic spline analyses assessed associations with LAA and mortality. Boruta feature selection, exploratory Mendelian randomization (MR), and *post hoc* stress hyperglycemia ratio (SHR) sensitivity analyses were also performed.

**Results:**

Lower HGI was independently associated with higher LAA risk in both cohorts (MIMIC-IV: OR = 0.579, *p* < 0.001; clinical cohort: OR = 0.599, *p* < 0.001), with an L-shaped nonlinear pattern. In MIMIC-IV, lower HGI was also associated with higher in-hospital mortality (OR = 0.488, *p* < 0.001). HGI showed the highest Boruta feature importance among measured baseline variables. HGI and SHR were strongly inversely correlated in both cohorts (Spearman’s *ρ* = −0.711 and −0.723; both *p* < 0.001), and higher SHR showed directionally consistent associations with adverse outcomes. MR did not provide significant genetic evidence linking HGI to LAA.

**Conclusion:**

Low HGI was associated with higher LAA risk and in-hospital mortality in IS. This signal overlaps with stress-hyperglycemia-related FPG-HbA1c discordance; therefore, HGI may be useful for risk stratification but should not be interpreted as an independent causal glycation phenotype.

## 1 Introduction

Stroke is currently the leading cause of death and long-term disability worldwide, of which ischemic stroke (IS) is the most common. Recent global burden of disease (GBD) analyses further highlight the enormous and growing threat of stroke to public health. Therefore, optimizing risk stratification for preventable stroke subtypes and delving deeper into their pathogenetic mechanisms has become an urgent priority (1).

IS has markedly heterogeneous etiology. Currently, the most well-known standardized subtype classification system for IS the Trial of Org 10,172 in Acute Stroke Treatment (TOAST) classification (2). IS can be divided into five main subtypes according to its main pathogenetic mechanism: (1) large-artery atherosclerosis (LAA), (2) cardioembolism (CE), (3) small-vessel occlusion (lacunar), (4) stroke of other determined etiology, and (5) stroke of undetermined etiology. It should be emphasized that etiological classification is not only used to describe disease conditions, but it also provides a direct basis for guiding clinical diagnosis and the development of secondary prevention strategies. The current American Heart Association/American Stroke Association (AHA/ASA) guidelines clearly indicate that individualized prevention strategies should be developed on the basis of different IS subtypes, the etiological evaluation should be strengthened, and the recurrence rate should be reduced by identifying interventional targets (3). Among these subtypes, the clinical significance of LAA is particularly important. LAA not only involves the occurrence of systemic atherosclerotic lesions but is also the focus of intensified risk factor control and vascular intervention when the indications are met.

Abnormal blood glucose metabolism and diabetes are currently recognized as important risk factors for cerebrovascular diseases. Glycated hemoglobin (HbA1c) is widely used clinically to assess long-term blood glucose exposure levels, and several population-based cohort studies have confirmed that the HbA1c level is closely related to the risk of IS (4). However, the HbA1c level does not perfectly reflect the true average blood glucose level in individuals. Owing to differences in biological characteristics and analytical methods (including differences in erythrocyte kinetics and glycation propensity among individuals), the actual measured HbA1c level may deviate from the expected value calculated based on the measured blood glucose level. If the HbA1c levels are interpreted in isolation, this “mismatch” between indicators may lead to clinical misjudgment (5).

To quantify this inconsistency, some scholars have proposed the use of the hemoglobin glycation index (HGI). The HGI is defined as the difference between an individual’s measured HbA1c level and the expected HbA1c level (predicted by a population regression model based on blood glucose levels in the same period), which represents the residual HbA1c level resulting from factors other than those contributing to the measured blood glucose level (6). Similarly, the “glycation gap” refers to the difference between the HbA1c level and the levels of other blood glucose biomarkers (such as fructosamines) to explain the systematic deviation in the glycation process (7). Increasing evidence indicates that such differences are not simply random errors. The glycation gap is temporally stable, suggesting a fixed underlying biological phenotype (8). In addition, inconsistencies in glycation indicators have been reported to correlate not only with microvascular complications but also with broader macrovascular events and mortality outcomes. This further supports the fact that the inherent “glycation tendency” of an individual may contain important clinical information independent of average blood glucose levels (9). Moreover, the practical value of the HGI has been demonstrated in large-scale clinical trials. For example, in the Action to Control Cardiovascular Risk in Diabetes (ACCORD) trial (10), the HGI was successfully used to identify subgroups that benefited from or experienced different degrees of damage from intensive hypoglycemic therapy, suggesting that the glycation phenotype may regulate vascular risk and treatment response in patients.

Although some progress has been made with regard to the HGI, the relationship between the HGI and cerebrovascular diseases (especially the specific pathogenesis of IS) is still unclear. The key to the precise prevention of IS is to understand the biological characteristics contributing to different stroke subtypes. However, previous studies on HbA1c indicators have largely focused on the overall incidence or overall prognosis of stroke and have rarely delved into specific etiological subtypes. Considering that LAA is a type of atherosclerosis accompanied by the diagnosis of severe metabolic disorders, we speculated that since the HGI can reflect an individual’s “glycation tendency” independent of blood glucose levels, it is likely to be closely related to the probability of LAA in patients experiencing IS. Understanding this relationship will not only aid in improving clinical classification and risk assessment but will also provide new theoretical insights into how glycation processes contribute to macrovascular diseases.

However, traditional observational studies are susceptible to interference from confounding factors and reverse causality. Especially in retrospective clinical cohorts, variables such as the patient’s underlying disease burden, the severity of the acute phase of the disease, the treatment intensity, and the detection frequency of various indicators often lead to bias in the correlation analysis. To account for this, the Mendelian randomization (MR) method provides a complementary framework for genetic epidemiological analysis. To satisfy specific assumptions, this method uses germline genetic variation as an instrumental variable, which greatly improves the reliability of causal inference. Previous studies that used MR have confirmed that diabetes and blood glucose-related characteristics are clearly causally correlated with cerebrovascular diseases. On this basis, when assessing the relationship between blood glucose-related exposures and specific stroke phenotypes, an MR-based triangulation strategy (11) was introduced in this study to obtain a more robust conclusion.

In summary, two independent clinical cohorts [the Medical Information Mart for Intensive Care-IV (MIMIC-IV) intensive care database (12) and an external neurology clinical cohort] were combined to specifically analyze the correlation between the HGI in patients experiencing IS and their risk of developing LAA. Moreover, to compensate for the deficiencies of traditional retrospective analyses in causality, this study also applied the MR method to explore potential causal relationships between glycation parameters and LAA using genetic variation, thereby strengthening the rigor and robustness of the evidence chain throughout the study.

## 2 Methods

### 2.1 Data sources

In this retrospective study, data from two independent cohorts were used: One was a large-scale public intensive care database (MIMIC-IV) and the other was a local clinical cohort from the Wuxi Traditional Chinese Medicine Hospital affiliated with the Nanjing University of Chinese Medicine.

#### 2.1.1 MIMIC-IV database

In this study, data from the MIMIC-IV database (version 3.1) was used (12). The use of the database was approved by the institutional review boards (IRBs) of the Massachusetts Institute of Technology (MIT) and the Beth Israel Deaconess Medical Center. Since the data used have been deidentified, the informed consent requirement was waived (13). All authenticated users completed the required training and signed a data usage agreement before being granted access. In this study, the principles of the *Declaration of Helsinki* (2024 revision) were followed (14). Researcher Chuxin Lyu completed relevant training as needed and signed a data use agreement to officially obtain access authorization to the MIMIC-IV database (Certificate Number: 61738903).

#### 2.1.2 Local hospital dataset

For the external validation cohort, the clinical data of patients experiencing IS who were hospitalized in the Department of Brain Diseases, Wuxi Traditional Chinese Medicine Hospital affiliated with Nanjing University of Chinese Medicine, between January 2024 and December 2025 were retrospectively included. This study was approved by the Ethics Committee of the Wuxi Traditional Chinese Medicine Hospital (Approval Number: 2024–087-02). Because this study was retrospective and the data were anonymized, a waiver of the informed consent requirement was granted.

### 2.2 Study population

In this study, patients hospitalized following an IS event were screened from the MIMIC-IV database using the ICD-9 and ICD-10 diagnosis codes (15) (see Supplementary Table S1 for a complete list of codes). In terms of the inclusion criteria, this study did not require IS to be the main cause of admission or a new disease; patients who were admitted for other diseases but were diagnosed with IS during hospitalization were also eligible for inclusion. In addition, for patients with multiple hospitalization records, only the data from their first admission were extracted for analysis.

**Figure 1:**
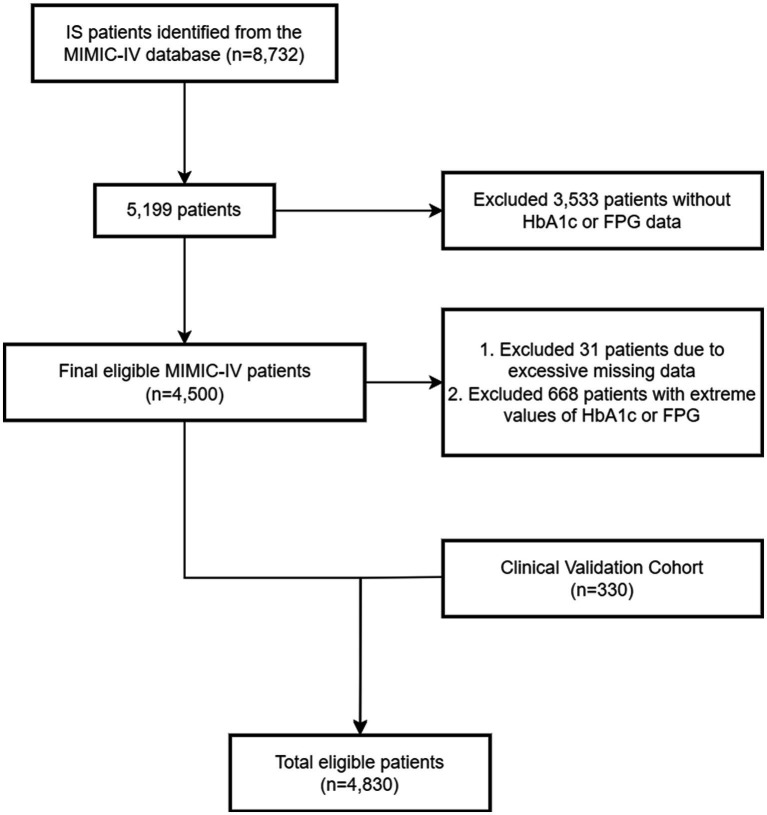
Flowchart detailing the study population selection process for both the MIMIC-IV database and the clinical validation cohort.

The inclusion criterion was as follows (Figure 1):

Age ≥ 18 years at first hospitalization.

The exclusion criterion was as follows:

Missing fasting plasma glucose (FPG) or HbA1c data.

The rate of missing data for key baseline indicators (including demographic characteristics, past medical history, laboratory test results, and outcome indicators) exceeded 25% (i.e., data integrity < 75%).

The data were outside the reasonable physiological range (that is, the measured value deviated from the mean by more than 4 standard deviations (SDs), or the value was an abnormal value incompatible with survival).

### 2.3 Data extraction and definitions

**Figure 2:**
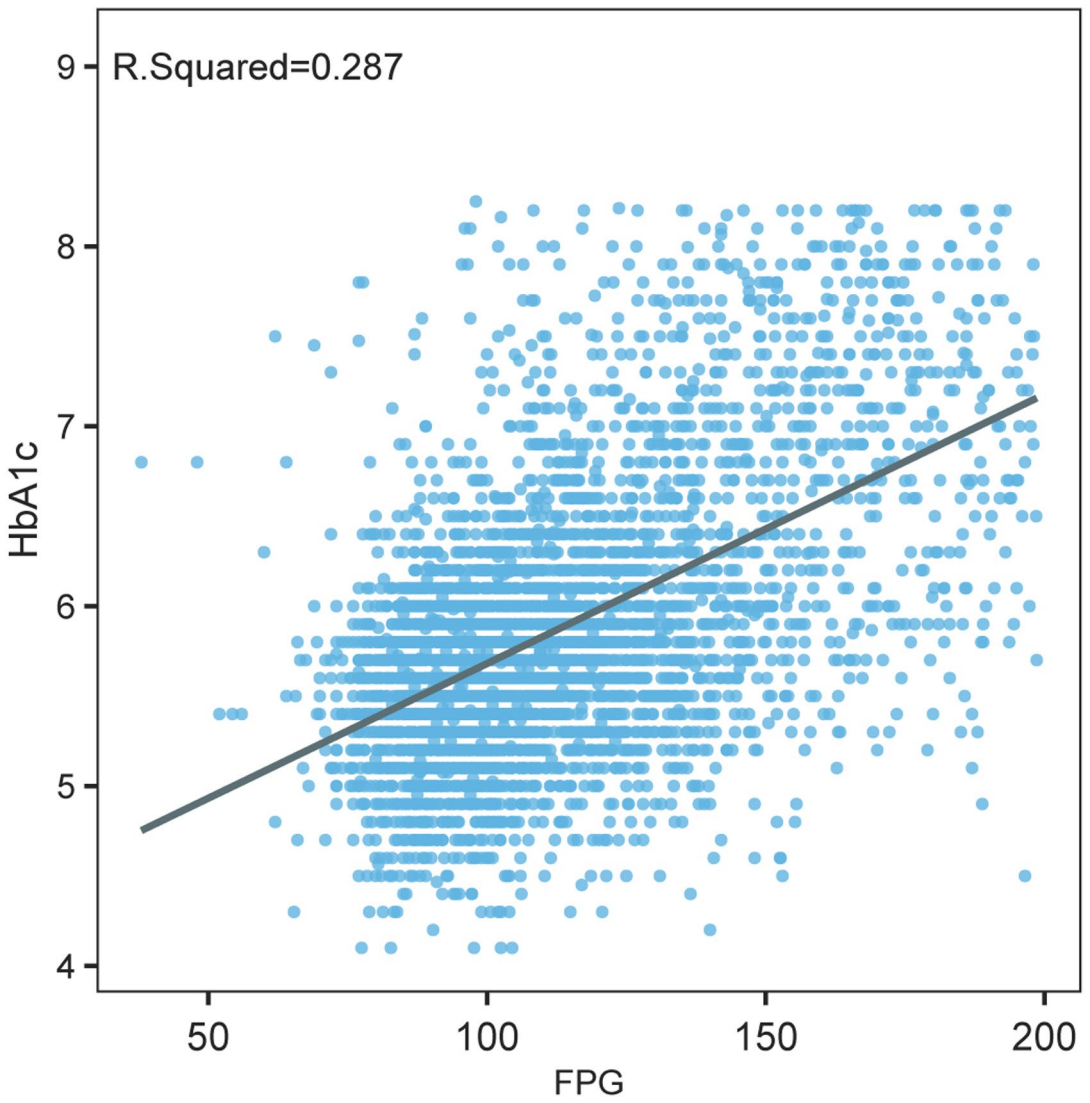
Relationship between the hemoglobin glycation index and measured HbA1c levels in the MIMIC-IV cohort.

**Figure 3:**
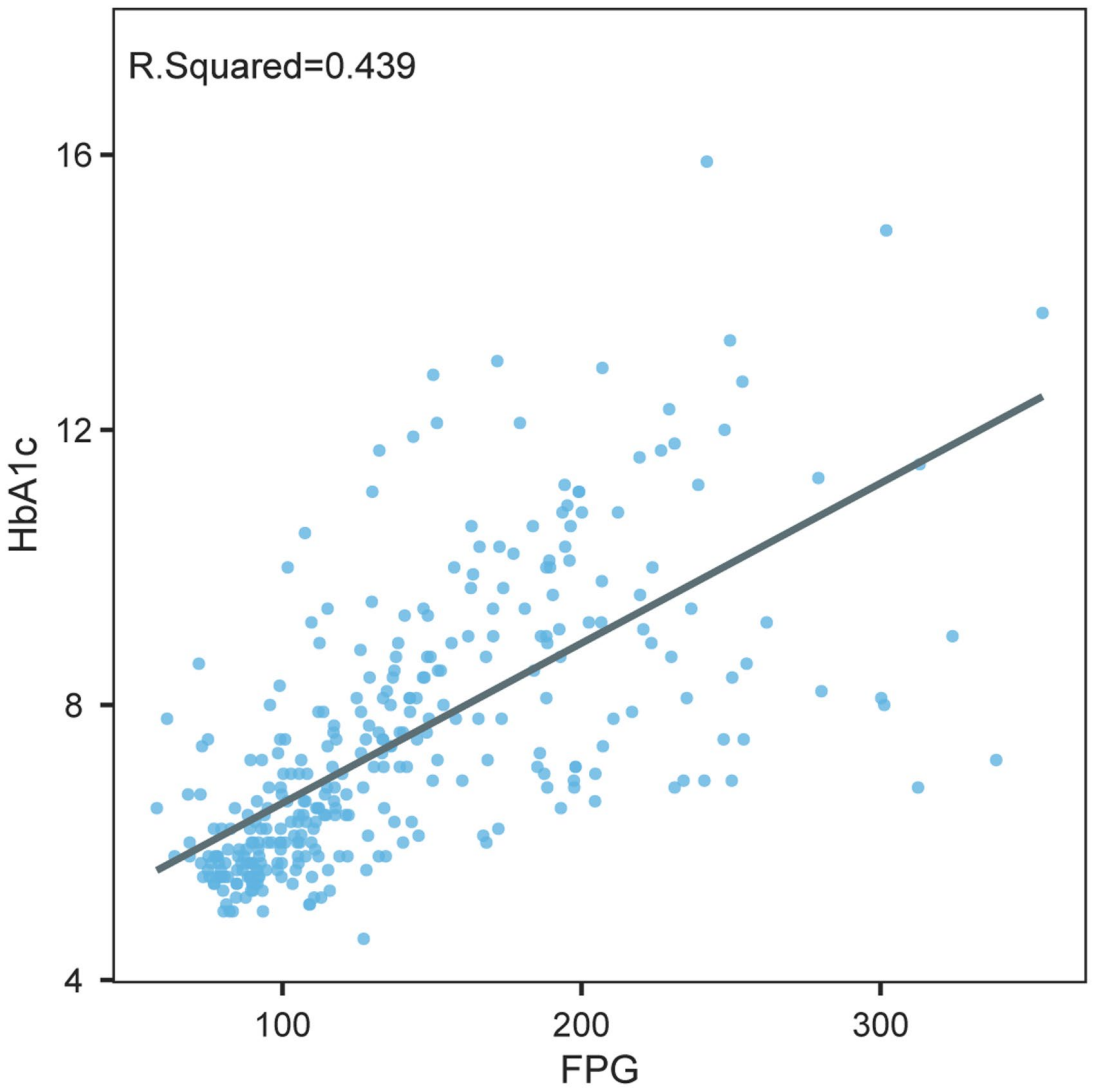
Relationship between the hemoglobin glycation index and measured HbA1c levels in the clinical validation cohort.

The baseline data of patients were extracted from the MIMIC-IV database using PostgreSQL software. Potential confounding variables included demographic characteristics (age, gender), comorbidities [hypertension (HTN), chronic kidney disease (CKD), atrial fibrillation (AF), ischemic heart disease (IHD), and type 2 diabetes mellitus (T2DM)], and laboratory indicators [platelets (PLTs), hemoglobin (Hb), red blood cells (RBCs), white blood cells (WBCs), triglycerides (TGs), creatinine (Cr), blood urea nitrogen (BUN), FPG, HbA1c, total cholesterol (TC), low-density lipoprotein (LDL), and high-density lipoprotein (HDL)]. For all laboratory indicators, the first recorded values after admission were used. In the MIMIC-IV cohort, laboratory timing was based on electronic timestamps, and whether FPG was obtained before all treatment interventions could not be fully verified. In the clinical validation cohort, fasting laboratory measurements were extracted from the first available post-admission blood tests recorded in the electronic medical record system. For the clinical validation cohort, demographic, clinical, and laboratory data were retrieved from the electronic medical records system of the Wuxi Traditional Chinese Medicine Hospital affiliated with the Nanjing University of Chinese Medicine. To ensure consistency between the two cohorts, HGI values were calculated using cohort-specific FPG–HbA1c regression equations according to the method described by Hempe et al. (10). Linear regression models were constructed separately in the MIMIC-IV cohort and the clinical validation cohort, with FPG as the independent variable and measured HbA1c as the dependent variable. The predicted HbA1c values were then calculated using the following equations: MIMIC-IV cohort, predicted HbA1c = 0.0150 × FPG + 4.1835; clinical validation cohort, predicted HbA1c = 0.0233 × FPG + 4.2459. The HGI was defined as the difference between the measured HbA1c level and the predicted level. In this study, analyses were performed at two levels. First, Stroke subtype classification was based on the TOAST framework. For the clinical validation cohort, TOAST subtype was retrospectively determined using available clinical records, neuroimaging findings, vascular imaging, electrocardiography, echocardiography, and other etiological evaluations recorded during hospitalization. For the MIMIC-IV cohort, because prospective TOAST adjudication and complete imaging information were not uniformly available, subtype information should be interpreted as diagnosis-code- and record-based classification rather than prospective TOAST adjudication. Second, the primary clinical endpoint was defined as in-hospital mortality, that is, all-cause death of patients during hospitalization. The relationships between the HGI and the HbA1c levels are shown in Figures 2, 3.

### 2.4 MR analysis

In this study, the two-sample MR method was used to investigate the genetic association between the genetically predicted HGI and the LAA risk of patients experiencing IS.

The genetic instruments used for predicting the HGI were obtained from the published genome-wide association study (GWAS) results of the ACCORD trial (16), wherein 24 independent dominant single nucleotide polymorphisms (SNPs) were identified at the *p* < 5 × 10^−6^ level. After excluding insertions and retaining only single nucleotide variations (SNVs) with *F* value > 10 (range: 12.25–49.00), 17 SNPs were ultimately selected as instrumental variables. To ensure independence between the instrumental variables, a strict threshold (*r*^2^ < 0.001 in a 10,000 kb window) was used to perform linkage disequilibrium (LD) cluster analysis.

Summary statistics of LAA were obtained from the MEGASTROKE consortium (OpenGWAS ID: ebi-a-GCST005840; *N* = 410,484). In addition, to evaluate subtype specificity, data on CE stroke (CES; ebi-a-GCST005842) and lacunar stroke [small vessel stroke (SVS); ebi-a-GCST005841] were extracted.

### 2.5 Statistical analysis

Categorical variables are expressed as numbers of cases (percentages) [n (%)]. Continuous variables were first tested for normality. Normally distributed variables are expressed as the mean ± standard deviation (mean ± SD), and nonnormally distributed variables are expressed as the median (interquartile range) [median (IQR)]. Intergroup comparisons of continuous variables were performed using Student’s t test or the Mann–Whitney U test. Categorical variables were analyzed using the chi-square test or Fisher’s exact test. The missing rates for each indicator were as follows: PLT 0.2%, TG 18.1%, TC 18.9%, LDL 21.5%, and HDL 19.6%. Missing covariate data were handled using multiple imputation by chained equations.

To investigate the independent correlation between the HGI and the IS LAA subtype, multivariate logistic regression models were constructed for data extracted from both the MIMIC-IV database and the clinical validation cohort. Three models were constructed to gradually adjust for confounding factors: Model 1 was unadjusted for the univariate analysis, Model 2 was adjusted for demographic characteristics (age and gender) and comorbidities (HTN, CKD, AF, IHD, and T2DM), and in Model 3, in addition to the adjustments in Model 2, PLT, Hb, RBC, WBC, TG, Cr, BUN, TC, LDL, and HDL were adjusted. The strength of association was reported as the odds ratio (OR) and 95% confidence interval (CI), and the restricted cubic spline (RCS) model was used to evaluate the potential nonlinear dose–response relationship between the HGI and the LAA subtype.

For the MIMIC-IV cohort, the Boruta algorithm was further used to screen the key features associated with LAA risk and subgroup analysis was performed based on the comorbidities to verify the robustness of the results. In addition, to assess the prognostic value of the HGI for all-cause in-hospital mortality in patients experiencing IS, three logistic regression models were used with the same correction strategy for covariates in the MIMIC-IV cohort and combined with RCS analysis to visualize the association between the HGI and the risk of death. All the statistical analyses were performed using R software (version 4.4.1). All the statistical tests were two-sided, and *p* < 0.05 was considered to indicate statistical significance.

The two-sample MR method employed the inverse variance weighted (IVW) regression method of the multiplicative random effects model to estimate the main causal effects. To assess the robustness of the results under different pleiotropic assumptions, the MR-Egger regression method, the weighted median method and the weighted mode method were used as supplementary analysis methods. Sensitivity analyses included Cochran’s Q test for assessing heterogeneity, the MR-Egger intercept test for testing horizontal pleiotropy, the Steiger filter and the leave-one-out method for confirming the direction of causality, and the MR-Pleiotropy RESidual Sum and Outlier (MR-PRESSO) method for detecting outliers. All the statistical analyses were performed using R software (version 4.4.1). The R packages used included TwoSampleMR, ieugwasr, MRPRESSO, and mr.raps.

To address the potential overlap between HGI and stress hyperglycemia, we performed additional *post hoc* sensitivity analyses using the stress hyperglycemia ratio (SHR). Estimated average glucose (eAG) was calculated from HbA1c as eAG = 28.7 × HbA1c − 46.7, and SHR was defined as FPG/eAG. Because HGI and SHR are both derived from FPG and HbA1c and are therefore mathematically coupled, they were not entered simultaneously into the same multivariable models. Instead, SHR was evaluated in parallel logistic regression models using the same covariate-adjustment strategy as the fully adjusted HGI analyses. Spearman correlation analyses were also performed separately in the MIMIC-IV cohort and the clinical validation cohort to quantify the overlap between HGI and SHR.

## 3 Results

### 3.1 Baseline patient characteristics

A total of 4,830 patients experiencing IS were included in this study (4,500 patients from the MIMIC-IV database and 330 patients from the clinical validation cohort). Tables 1, 2 provide a summary of the overall baseline characteristics and stratification of patients according to the LAA subtype. In the MIMIC-IV cohort, patients in the LAA group were older, and AF and CKD were more common; the gender ratio and the prevalence of HTN, T2DM and IHD were similar between the two groups. There were also significant differences in laboratory indicators; the WBCs and FBG levels of patients in the LAA group were higher, and the Hb, TG, BUN, TC, LDL, and HbA1c levels were lower. In the clinical validation cohort, the results of key glucose, lipid metabolism, and inflammatory markers exhibited similar trends; patients in the LAA group had significantly higher levels of WBCs, FBG, LDL, and HDL, while the prevalence of various complications was not significantly different between the two groups. Notably, in both independent cohorts, the core study endpoint, the HGI value, was significantly lower in patients in the LAA group compared with those in the non-LAA group (MIMIC-IV cohort: −0.19 vs. −0.019, *p* < 0.001; clinical cohort: −0.539 vs. −0.038, p < 0.001); while other conventional blood parameters, such as PLTs, RBCs, and Cr levels, remained comparable between the two groups in both cohorts. The FPG–HbA1c regression models showed moderate explanatory ability. The *R*^2^ values were 0.287 in the MIMIC-IV cohort and 0.439 in the clinical validation cohort, as shown in Figures 2, 3. These values indicate that HGI contains a substantial residual component beyond FPG-predicted HbA1c, which may reflect interindividual glycation tendency, erythrocyte biology, measurement variability, or acute FPG–HbA1c discordance.

**Table 1:** Baseline characteristics of patients with ischemic stroke in the MIMIC-IV database stratified by the presence of large-artery atherosclerosis.

**Variables**	**Overall**	**Non-LAA**	**LAA**	***P* value**
*n*	4,500	3,725	775	
Age, years	73 (18–101)	73 (18–101)	75 (24–101)	<0.001
PLT, K/uL	212 (10–1,479)	212 (10–1,479)	213 (15–708)	0.371
Hb, g/dl	12.3 (4.1–18.6)	12.3 (4.1–18.6)	12 (5.9–18.3)	0.012
RBC, g/dl	4.10 (1.16–8.58)	4.11 (1.16–8.58)	4.05 (1.89–6.13)	0.091
WBC, g/dl	8.3 (0.1–235)	8 (0.1–228.6)	9.4 (0.9–235)	<0.001
TG, mg/dL	108 (24–2,786)	111 (24–2016)	97 (30–2,786)	0.031
Cr, mg/dL	0.9 (0.3–15.5)	0.9 (0.3–15.5)	0.9 (0.3–12.4)	0.256
BUN, mg/dL	17 (3–189)	17 (3–189)	16 (4–149)	0.043
TC, mg/dL	159 (42–486)	160 (44–486)	157 (42–355)	0.004
LDL, mg/dL	86 (3–378)	87 (3–378)	83 (5–268)	0.013
HDL, mg/dL	45 (5–137)	45 (5–137)	46 (11–126)	0.194
FBG, mg/dL	104.775 (38–198.6)	103.667 (38–198)	110.5 (62–198.6)	<0.001
HbA1c, %	5.7 (4.1–8.25)	5.7 (4.1–8.25)	5.7 (4.1–8.213)	0.017
HGI	−0.041 (−2.631–2.596)	−0.019 (−2.631–2.596)	−0.19 (−1.874–2.232)	<0.001
Male, *n* (%)	2,247 (49.93)	1881 (50.50)	366 (47.23)	0.106
AF, *n* (%)	1,456 (32.36)	1,158 (31.09)	298 (38.45)	<0.001
HTN, *n* (%)	2,452 (54.49)	2051 (55.06)	401 (51.74)	0.099
T2DM, *n* (%)	1,151 (25.58)	954 (25.61)	197 (25.42)	0.947
CKD, *n* (%)	754 (16.76)	603 (16.19)	151 (19.48)	0.029
IHD, *n* (%)	1,261 (28.02)	1,039 (27.89)	222 (28.65)	0.704

**Table 2:** Baseline characteristics of patients with ischemic stroke in the clinical validation cohort stratified by the presence of large-artery atherosclerosis.

**Variables**	**Overall**	**Non-LAA**	**LAA**	***P* value**
*n*	330	231	99	
Age, years	71 (31–96)	71 (31–96)	74 (38–94)	0.167
PLT, K/uL	188.5 (24–563)	189 (24–563)	188 (96–365)	0.524
Hb, g/dl	14.2 (1.58–19.7)	14.2 (1.58–17.9)	14.3 (7.9–19.7)	0.642
RBC, g/dl	4.645 (2.67–10.39)	4.64 (2.67–6.62)	4.69 (3.14–10.39)	0.474
WBC, g/dl	7.01 (3.34–18.34)	6.9 (3.34–18.21)	7.24 (4.03–18.34)	0.041
TG, mg/dL	189.92 (40.53–2743.5)	185.85 (40.53–2743.5)	246.92 (51.51–681.45)	0.448
Cr, mg/dL	0.82 (0.30–3.96)	0.84 (0.30–2.68)	0.78 (0.46–3.96)	0.609
BUN, mg/dL	16.52 (3.39–74.48)	16.24 (3.39–74.48)	16.8 (7.28–60.76)	0.119
TC, mg/dL	169.91 (81.57–457.73)	170.49 (81.57–457.73)	168.94 (93.56–297.68)	0.162
LDL, mg/dL	95.88 (22.42–197.94)	94.72 (22.42–197.94)	99.74 (32.86–188.66)	0.046
HDL, mg/dL	41.37 (18.94–78.87)	41.37 (18.94–78.87)	43.30 (25.13–78.48)	0.008
FBG, mg/dL	120.61 (58.01–354.18)	116.74 (58.01–324.09)	133.67 (61.43–354.18)	0.001
HbA1c, %	6.9 (4.6–15.9)	6.8 (5–15.9)	7 (4.6–13.7)	0.196
HGI	−0.236 (−4.836–6.089)	−0.038 (−3.052–6.089)	−0.539 (−4.836–3.862)	<0.001
Male, *n* (%)	218 (66.06)	157 (67.97)	61 (61.62)	0.322
AF, *n* (%)	34 (10.30)	21 (9.09)	13 (13.13)	0.363
HTN, *n* (%)	258 (78.18)	176 (76.19)	82 (82.83)	0.233
T2DM, *n* (%)	169 (51.21)	123 (53.25)	46 (46.46)	0.313
CKD, *n* (%)	18 (5.45)	15 (6.49)	3 (3.03)	0.315
IHD, *n* (%)	37 (11.21)	22 (9.52)	15 (15.15)	0.196

### 3.2 Feature selection using the Boruta algorithm

**Figure 4:**
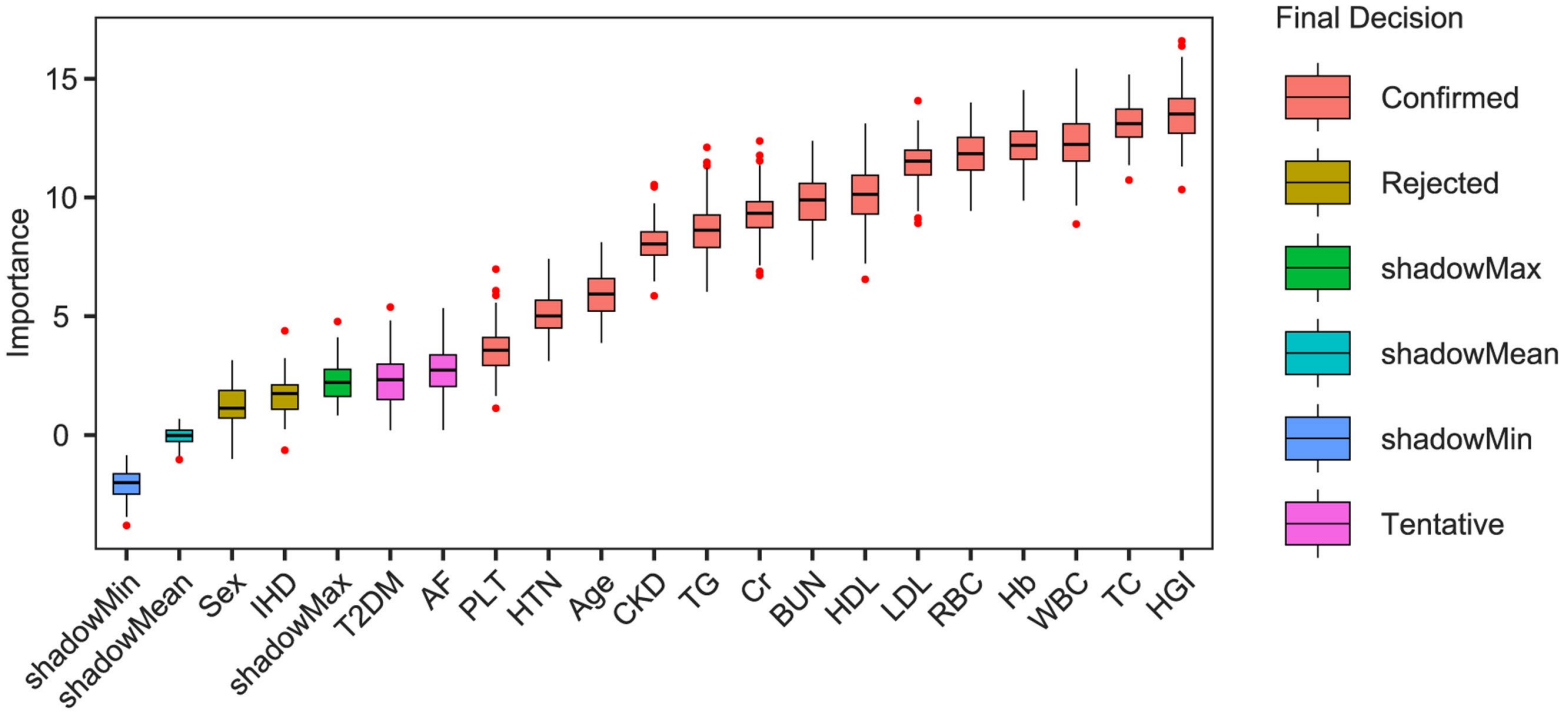
Feature selection using the Boruta algorithm to identify key baseline variables associated with the large-artery atherosclerosis subtype in the MIMIC-IV cohort.

To accurately identify the markers that were most closely related to the LAA subtype from the high-dimensional clinical data and minimize collinear interference in the subsequent multivariate analysis, the Boruta machine learning algorithm for feature selection was implemented in the MIMIC-IV cohort. As shown in Figure 4, the Boruta algorithm was used to evaluate the feature importance of each baseline variable based on the random forest model. After iterative calculation, a total of 14 variables were successfully determined as “confirmed” features with significant predictive value for LAA. The features were ranked from highest to lowest based on median importance as follows: HGI, TC, WBC, Hb, RBC, LDL, HDL, BUN, Cr, TG, CKD, Age, HTN, and PLT.

### 3.3 Association and nonlinear relationship between the HGI and LAA risk

To evaluate the independent correlation between the HGI and LAA risk, three stepwise-corrected multivariate logistic regression models were constructed for the MIMIC-IV cohort (Table 3) and the clinical validation cohort (Table 4). When the HGI was analyzed as a continuous variable, in Model 1, which was not adjusted for any covariates, the HGI was significantly negatively correlated with LAA risk (MIMIC-IV cohort: OR = 0.635, 95% CI: 0.556–0.725, *p* < 0.05; clinical cohort: OR = 0.644, 95% CI: 0.524–0.778, *p* < 0.001). After adjusting for age, gender and multiple comorbidities (Model 2) and further comprehensively adjusting for various laboratory indicators (Model 3), the independent negative correlation was still highly robust. In the fully adjusted Model 3, for every unit increase in the HGI, the LAA risk in the MIMIC-IV cohort and the clinical validation cohort was significantly reduced by 42.1% (OR = 0.579; 95% CI: 0.498–0.671; *p* < 0.001) and 40.1% (OR = 0.599; 95% CI: 0.465–0.755; *p* < 0.001), respectively. To further verify these findings and explore the dose–response relationship, the HGI was converted into quartiles (Q1–Q4) and reintroduced in the analysis. When the lowest quartile (Q1) was used as the reference group, with increasing HGI level, the LAA risk in both cohorts tended to decrease. For the MIMIC-IV cohort, when the fully adjusted Model 3 was used, compared with that of the Q1 group, the ORs of LAA in the Q2, Q3, and Q4 groups were 0.682, 0.499, and 0.454, respectively (all *p* values < 0.001), and the trend test demonstrated high statistical significance (*P* for trend < 0.001). Similarly, a completely consistent pattern was also observed in Model 3 for the clinical validation cohort. The LAA risk in the Q4 group was significantly lower than that in the Q1 group (OR = 0.313, 95% CI: 0.128–0.744; *p* = 0.009), and the trend test results were still significant (*P* for trend = 0.002).

**Table 3:** Multivariate logistic regression analysis of the association between the hemoglobin glycation index (HGI) and the risk of large-artery atherosclerosis (LAA) in the MIMIC-IV cohort.

**Variables**	**Model 1**		**Model 2**		**Model 3**	
	OR (95% CI)	*P*	OR (95% CI)	*P*	OR (95% CI)	*P*
HGI continuous	0.635 (0.556, 0.725)	<0.001	0.578 (0.501, 0.666)	<0.001	0.579 (0.498, 0.671)	<0.001
HGI group	*P* for trend: <0.001		*P* for trend: <0.001		*P* for trend: <0.001	
Q1	Ref		Ref		Ref	
Q2	0.686 (0.557, 0.842)	<0.001	0.686 (0.557, 0.844)	<0.001	0.682 (0.551, 0.842)	<0.001
Q3	0.528 (0.424, 0.655)	<0.001	0.505 (0.405, 0.629)	<0.001	0.499 (0.397, 0.625)	<0.001
Q4	0.512 (0.411, 0.637)	<0.001	0.451 (0.355, 0.572)	<0.001	0.454 (0.355, 0.579)	<0.001

Model 1: unadjusted; Model 2: adjusted for age, sex, HTN, CKD, T2DM, IHD, and AF; Model 3: adjusted for age, sex, HTN, CKD, T2DM, IHD, AF, PLT, Hb, RBC, WBC, TG, Cr, BUN, TC, LDL, HDL.

**Table 4:** Multivariate logistic regression analysis of the association between the hemoglobin glycation index (HGI) and the risk of large-artery atherosclerosis (LAA) in the clinical validation cohort.

**Variables**	**Model 1**		**Model 2**		**Model 3**	
	OR (95% CI)	*P*	OR (95% CI)	*P*	OR (95% CI)	*P*
HGI continuous	0.644 (0.524, 0.778)	<0.001	0.596 (0.469, 0.744)	<0.001	0.599 (0.465, 0.755)	<0.001
HGI group	*P* for trend: <0.001		*P* for trend: <0.001		*P* for trend: 0.002	
Q1	Ref		Ref		Ref	
Q2	0.367 (0.189, 0.698)	0.003	0.389 (0.197, 0.752)	0.006	0.437 (0.212, 0.886)	0.023
Q3	0.242 (0.118, 0.479)	<0.001	0.227 (0.106, 0.469)	<0.001	0.246 (0.109, 0.534)	<0.001
Q4	0.313 (0.159, 0.600)	<0.001	0.294 (0.131, 0.643)	0.003	0.313 (0.128, 0.744)	0.009

Model 1: unadjusted; Model 2: adjusted for age, sex, HTN, CKD, T2DM, IHD, and AF; Model 3: adjusted for age, sex, HTN, CKD, T2DM, IHD, AF, PLT, Hb, RBC, WBC, TG, Cr, BUN, TC, LDL, HDL.

**Figure 5:**
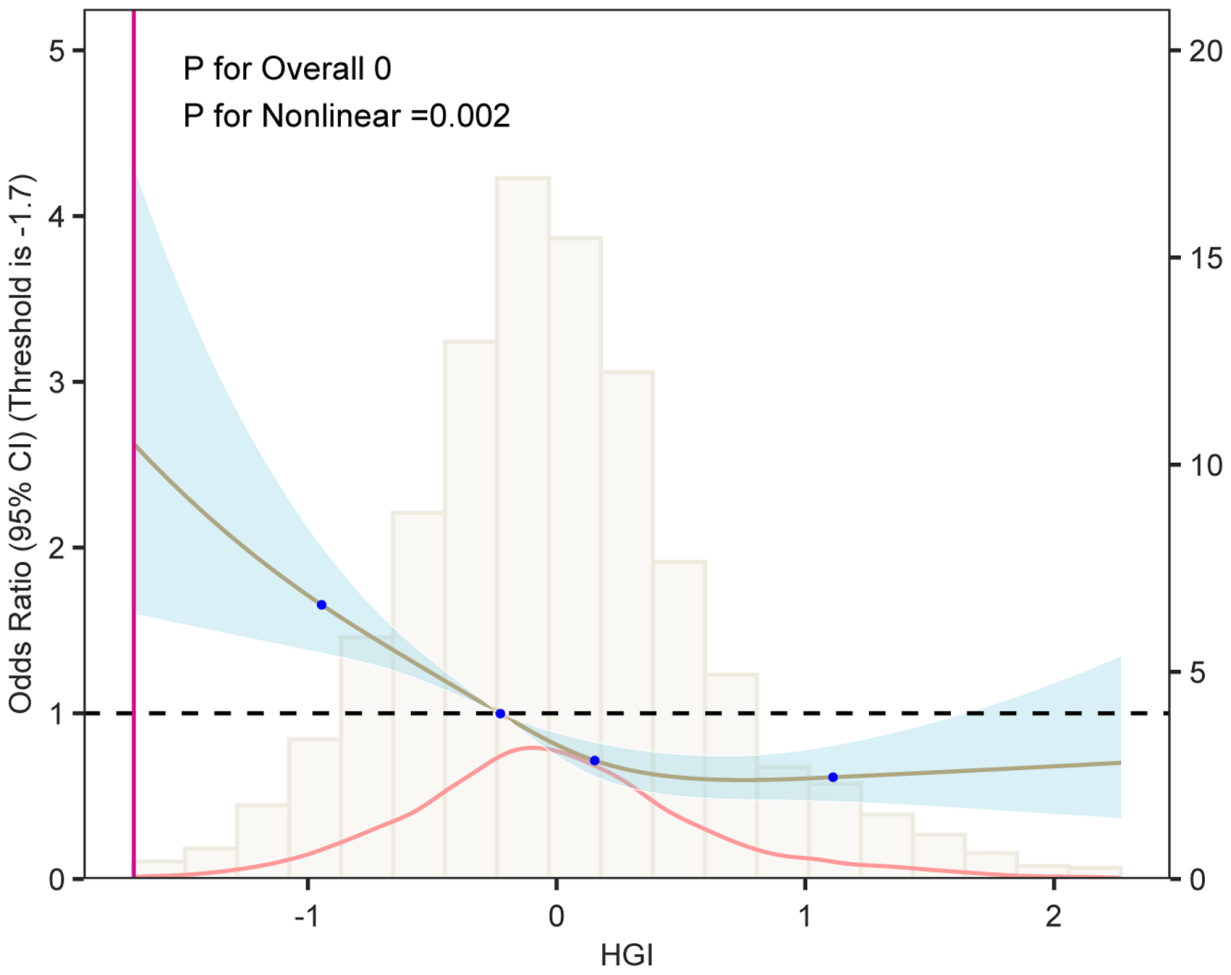
Restricted cubic spline curve illustrating the nonlinear association between HGI and the risk of large-artery atherosclerosis in the MIMIC-IV cohort. The spline was fitted using quantile-based knots according to the RCS model specification. The solid curve represents the adjusted odds ratio, the shaded area represents the 95% confidence interval, the dashed horizontal line indicates OR = 1, the vertical line indicates the reference value used in the model, and the histogram shows the distribution of HGI.

**Figure 6:**
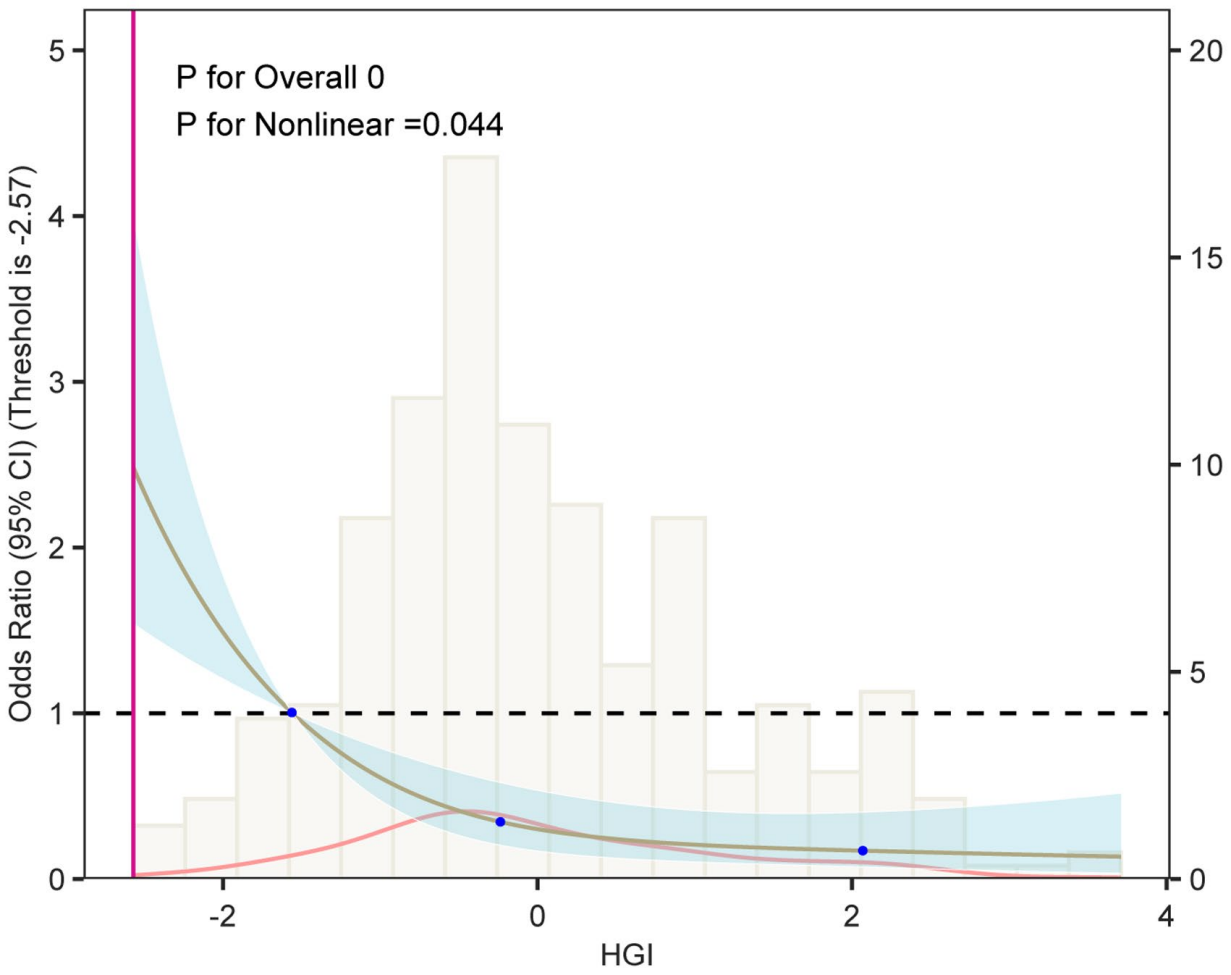
Restricted cubic spline curve illustrating the nonlinear association between HGI and the risk of large-artery atherosclerosis in the clinical validation cohort. The solid curve represents the adjusted odds ratio, the shaded area represents the 95% confidence interval, the dashed horizontal line indicates OR = 1, the vertical line indicates the reference value used in the model, and the histogram shows the distribution of HGI.

To further characterize the exact dose–response relationship, RCS analysis was combined with the fully adjusted model (Model 3). As shown in Figure 5, in the MIMIC-IV cohort, the RCS curve revealed a significant nonlinear association between the HGI and the LAA risk (overall *p* < 0.001, nonlinear *p* = 0.002). The overall curve showed a significant nonlinear downward trend: In the interval where the HGI was low, the LAA risk (measured by the OR) decreased relatively sharply with increasing HGI; after crossing a certain threshold, the decreasing trend of this risk gradually slowed and tended to stabilize, resulting in an L-shaped trajectory. This nonlinear dose–response pattern was reproduced in the clinical validation cohort (Figure 6). RCS analysis of the clinical cohort also confirmed a significant nonlinear negative correlation (overall p < 0.001, nonlinear *p* = 0.044), and the shape of the curve was highly consistent with that of the MIMIC-IV cohort, both of which showed a rapid decline in risk in the early period followed by a gradually flattening slope.

### 3.4 Association and nonlinear relationship between the HGI and in-hospital all-cause mortality

In addition to the primary outcome of the LAA subtype, the independent association between the HGI and all-cause mortality in patients experiencing IS in the MIMIC-IV cohort was further explored (Table 5). The HGI was included as a continuous variable in the stepwise adjusted multivariate logistic regression models. The results showed that HGI was negatively associated with the risk of in-hospital mortality. In Model 3, after adjustment for available demographic characteristics, comorbidities, and laboratory covariates, each unit increase in HGI was associated with a lower risk of in-hospital all-cause mortality (OR = 0.488; 95% CI: 0.379–0.628; *p* < 0.001). To assess the gradient effect of this association, the HGI values were converted to quartiles (Q1–Q4) for analysis. When the lowest quartile (Q1) was used as the reference group, as the HGI level increased, the mortality risk significantly decreased. In the fully adjusted Model 3, the ORs of in-hospital mortality in the Q2, Q3, and Q4 groups were 0.431 (95% CI: 0.277–0.654), 0.464 (95% CI: 0.299–0.705), and 0.373 (95% CI: 0.299–0.705), respectively, all *p* values were < 0.001, and there was a highly significant statistical trend (*P* for trend < 0.001).

**Table 5:** Multivariate logistic regression analysis of the association between the hemoglobin glycation index and the risk of all-cause in-hospital mortality in the MIMIC-IV cohort.

**Variables**	**Model 1**		**Model 2**		**Model 3**	
	OR (95% CI)	*P*	OR (95% CI)	*P*	OR (95% CI)	*P*
HGI continuous	0.488 (0.379, 0.628)	<0.001	0.475 (0.367, 0.613)	<0.001	0.488 (0.379, 0.628)	<0.001
HGI group	*P* for trend: 0.001		*P* for trend: <0.001		*P* for trend: <0.001	
Q1	Ref		Ref		Ref	
Q2	0.372 (0.245, 0.552)	<0.001	0.396 (0.259, 0.590)	<0.001	0.431 (0.277, 0.654)	<0.001
Q3	0.406 (0.271, 0.597)	<0.001	0.417 (0.277, 0.618)	<0.001	0.464 (0.299, 0.705)	<0.001
Q4	0.372 (0.245, 0.552)	<0.001	0.336 (0.214, 0.519)	<0.001	0.373 (0.233, 0.584)	<0.001

Model 1: unadjusted; Model 2: adjusted for age, sex, HTN, CKD, T2DM, IHD, and AF; Model 3: adjusted for age, sex, HTN, CKD, T2DM, IHD, AF, PLT, Hb, RBC, WBC, TG, Cr, BUN, TC, LDL, HDL.

**Figure 7:**
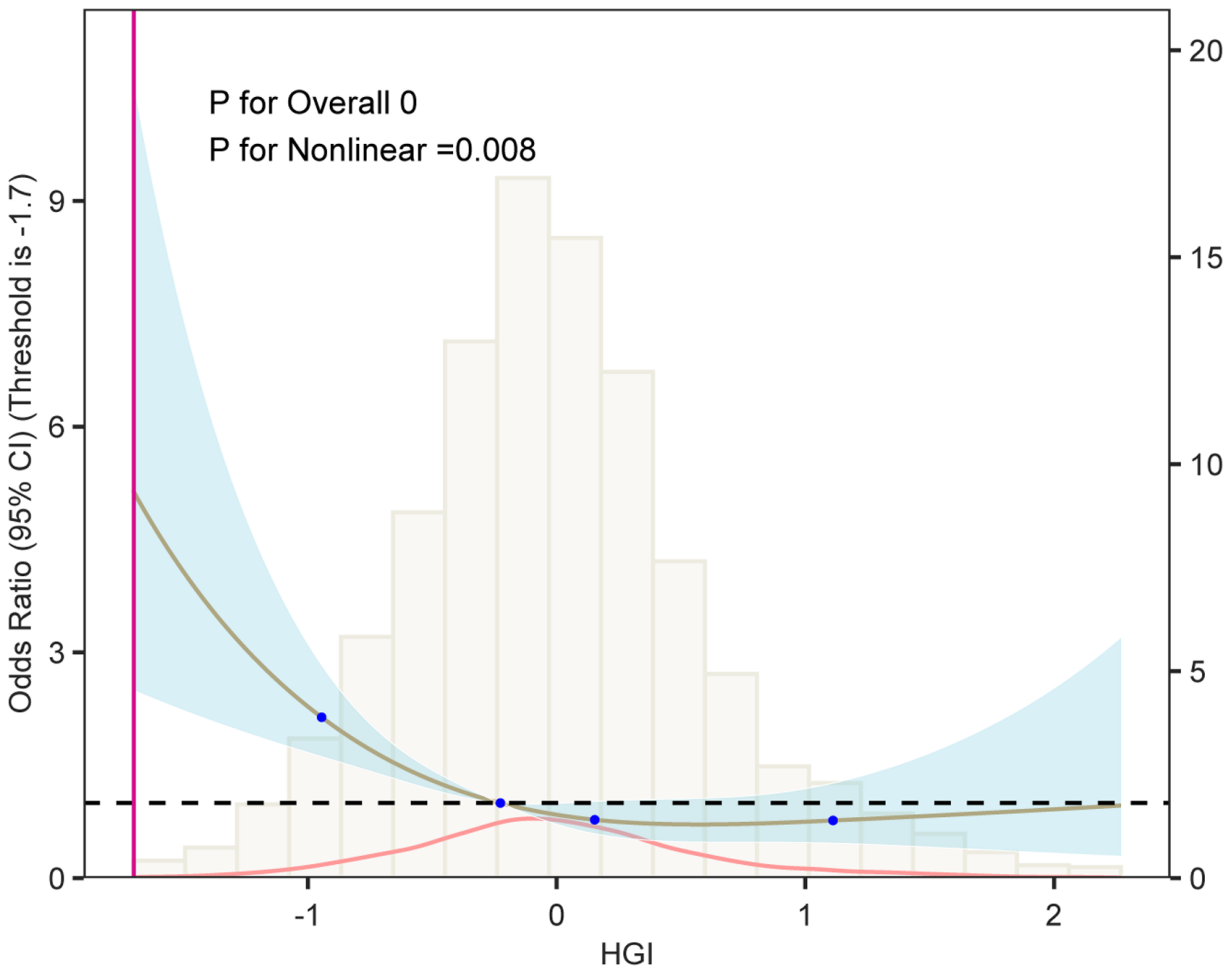
Restricted cubic spline curve illustrating the nonlinear association between HGI and the risk of all-cause in-hospital mortality in the MIMIC-IV cohort. The solid curve represents the adjusted odds ratio, the shaded area represents the 95% confidence interval, the dashed horizontal line indicates OR = 1, the vertical line indicates the reference value used in the model, and the histogram shows the distribution of HGI.

RCS analysis was performed to further examine the dose–response relationship between the HGI and all-cause in-hospital mortality. As shown in Figure 7, the RCS curve revealed a significant nonlinear correlation between the two parameters (overall *p* < 0.001, nonlinear *p* = 0.008). Consistent with the pattern of LAA risk, the RCS curve of the risk of all-cause mortality also showed a typical “L”-shaped decreasing trajectory. In the low-HGI interval, as the HGI increased, the risk of all-cause in-hospital mortality decreased sharply. When the HGI value exceeded a certain threshold, the marginal benefit of this protective effect gradually decreased, and the curve subsequently slowed and stabilized within the high HGI interval.

In the *post hoc* SHR sensitivity analysis, HGI and SHR were strongly inversely correlated in both cohorts (MIMIC-IV cohort: Spearman’s *ρ* = −0.711, *p* < 0.001; clinical validation cohort: Spearman’s ρ = −0.723, *p* < 0.001). When SHR was used instead of HGI in the fully adjusted models, higher SHR was associated with a higher risk of LAA in the MIMIC-IV cohort (OR = 6.287, 95% CI: 4.201–9.416, *p* < 0.001) and in the clinical validation cohort (OR = 13.957, 95% CI: 4.475–48.450, *p* < 0.001). In the MIMIC-IV cohort, higher SHR was also associated with increased in-hospital all-cause mortality (OR = 11.799, 95% CI: 6.053–22.984, *p* < 0.001). These findings indicate that the association observed for low HGI is directionally consistent with stress-hyperglycemia-related FPG–HbA1c discordance (Supplementary Table S2).

### 3.5 Subgroup analyses

**Figure 8:**
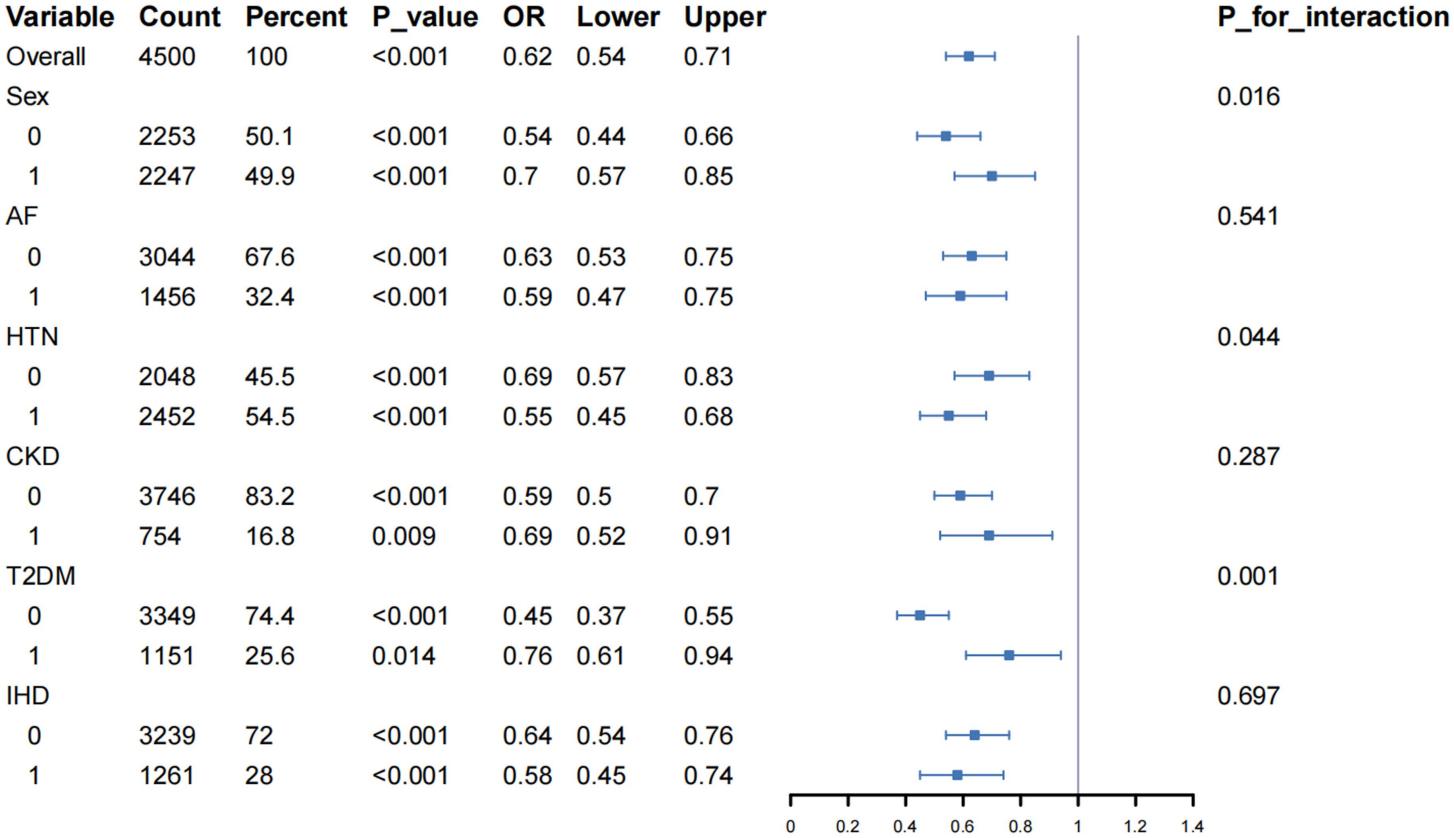
Forest plot of subgroup analyses assessing the robustness of the association between the hemoglobin glycation index and the risk of large-artery atherosclerosis stratified by sex and key comorbidities in the MIMIC-IV cohort.

To further evaluate the robustness of the negative correlation between the HGI and the LAA risk and explore the potential effects of population heterogeneity, a comprehensive stratified analysis was conducted for the MIMIC-IV cohort on the basis of sex and multiple key comorbidities (AF, HTN, CKD, T2DM, and IHD). As shown in the forest plot in Figure 8, there was a stable and significantly negative correlation between the HGI and LAA risk in all preset subgroups (the OR values of all the subgroups were less than 1, and the *p-*values were all <0.05). This finding indicates that regardless of whether patients have comorbidities, a relatively low HGI is always a robust risk factor for LAA.

It is worth noting that the interaction test revealed that some clinical characteristics significantly modified the effect of the HGI. Specifically, the degree of negative correlation between the HGI and LAA risk differed significantly among groups comprising patients with different genders (*P* for interaction = 0.016) with or without HTN (*P* for interaction = 0.044) and with or without T2DM (*P* for interaction = 0.001). Among them, in the population without T2DM (OR = 0.45, 95% CI: 0.37–0.55), the risk-reducing effect related to the HGI was significantly greater than that in patients with T2DM (OR = 0.76, 95% CI: 0.61–0.94). With regard to the stratification of patients based on AF, CKD, and IHD, no significant interaction was observed (all *p* values for interaction > 0.05), suggesting that the predictive value of the HGI in these different physiological backgrounds remained highly homogeneous.

### 3.6 MR analysis

**Figure 9:**
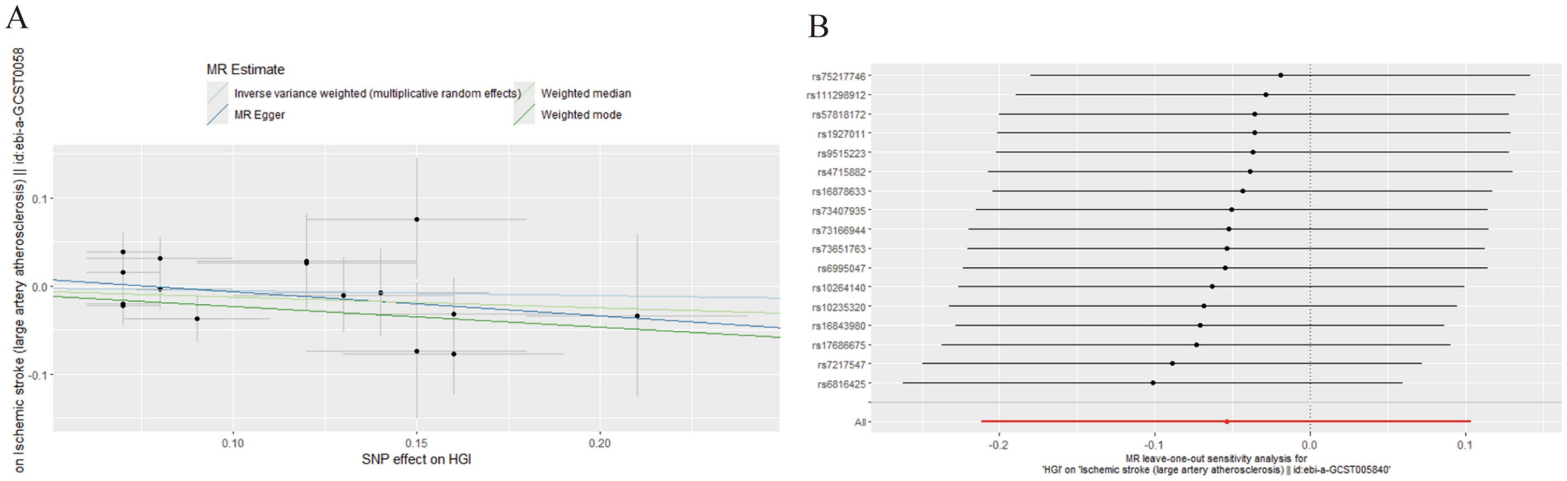
Mendelian randomization analysis assessing the genetic association between hemoglobin glycation index and the risk of large-artery atherosclerosis. **(A)** Scatter plot showing the effect sizes of individual single nucleotide polymorphisms on HGI against their corresponding effects on LAA risk. **(B)** Leave-one-out sensitivity analysis evaluating whether exclusion of any individual SNP materially changed the overall MR estimate.

To further evaluate the observational association from a genetic perspective, a two-sample MR analysis was performed. The primary IVW analysis did not provide statistically significant evidence for a causal effect of genetically predicted HGI on LAA risk (OR = 0.95, 95% CI: 0.81–1.11; *p* = 0.50). Although the point estimates from IVW, MR-Egger, weighted median, and weighted mode were directionally negative, the confidence intervals crossed the null value. Therefore, these MR findings should be interpreted as exploratory rather than confirmatory. The leave-one-out analysis did not identify any single SNP that materially dominated the overall estimate; however, this result does not establish statistical significance or prove causality (Figure 9A) (combined scatter plot and leave-one-out sensitivity analysis also confirmed the robustness of the trend, and no single-SNP driver effect was observed) (Figure 9B). In contrast, this consistency was not observed for the other IS subtypes. For CES, the IVW estimate was close to the null value (OR = 1.02, 95% CI: 0.87–1.19; *p* = 0.81). Similarly, for SVS, the IVW estimate was also close to the null value (OR = 1.03, 95% CI: 0.92–1.15; *p* = 0.59), suggesting no clear genetic evidence for an association between HGI and these stroke subtypes in the current dataset.

## 4 Discussion

In this study, the association between the HGI and LAA in patients experiencing IS was systematically assessed in two independent populations. The multivariate logistic regression results of the two cohorts revealed that the HGI was stably negatively correlated with the occurrence of LAA (MIMIC-IV cohort: OR = 0.579, 95% CI 0.459–0.729; our hospital: OR = 0.599, 95% CI 0.463–0.729; both *p* < 0.001), and the RCS analysis revealed an L-shaped nonlinear relationship (nonlinear *p* = 0.002; threshold: approximately −0.19). Moreover, the HGI was negatively correlated with the risk of in-hospital all-cause mortality, and a nonlinear pattern was detected (multivariate logistic analysis: OR = 0.488, 95% CI 0.285–0.835, *p* = 0.009; nonlinear *p* = 0.008; threshold: approximately −0.10). At the feature-selection level, the Boruta algorithm suggested that HGI had the highest predictive importance among the measured baseline variables. However, machine-learning feature importance reflects contribution to model classification and does not imply causal or biological importance.

Previous studies on the HGI have focused mainly on the prediction of the risk of cardiovascular diseases and mortality outcomes. In recent years, increasing evidence has suggested that the relationship between the HGI and adverse outcomes is not simply linear, and “low-HGI risk” is a consistent outcome in many scenarios. For example, in a study of diabetes mellitus patients with coronary heart disease with a large sample size (17), there was a U-shaped relationship between the HGI and major adverse cardiovascular events (MACEs), and the risk of all-cause mortality and cardiovascular death was greater in people with a low HGI. A U-shaped relationship between the HGI and all-cause/cardiovascular mortality has also been observed in the general population during long-term follow-up (18). In the MIMIC-IV study on patients with critical coronary heart disease (19), a low HGI was significantly correlated with short-term and long-term mortality risks, exhibiting a U-shaped nonlinear correlation.

It is worth emphasizing that evidence regarding the HGI from the stroke population has also accumulated rapidly in recent years. A study on patients with acute IS conducted using the MIMIC-IV database (20) reported that the HGI and short-term mortality risk could have an L-shaped or similar nonlinear pattern, and a low HGI was consistently associated with worse short-term outcomes. Another study that used data from the MIMIC-IV 2.2 database (21) suggested a differential association between the HGI and short-term/medium-term and long-term mortality in critically ill patients with acute IS, supporting the general direction of “nonlinear + low risk”. In addition, for patients with large vessel occlusion of the anterior circulation who underwent endovascular treatment (EVT), the HGI showed a U-shaped relationship with 90-day adverse functional outcomes, and a low HGI was significantly associated with adverse outcomes (22).

Analysis of baseline characteristics revealed that the HGI of patients in the LAA group was lower than that in the non-LAA group. Moreover, this group of patients also exhibited a unique combination of indicators; that is, fasting blood glucose levels and white blood cell counts were high, and HbA1c levels were low. From the perspective of the composition of the indicators, HGI is the “observed HbA1c − predicted HbA1c (obtained by FPG regression)”; therefore, when patients have an elevated blood glucose level at admission or in the acute phase and when the HbA1c level does not increase synchronously, the HGI is more likely to be negative or decrease. Conceptually, this is highly consistent with “stress hyperglycemia” or “relative hyperglycemia”: In the context of acute diseases, the body’s counterregulatory hormones and inflammatory responses can rapidly disturb stable glucose homeostasis, resulting in a short-term increase in the blood glucose level; as a long-term window indicator, it is difficult for the HbA1c level to reflect this acute fluctuation synchronously.

Numerous studies in recent years (23, 24) have reported that the formula: stress hyperglycemia ratio (SHR) = blood glucose level at admission/mean blood glucose level calculated from the HbA1c level” can be used to characterize relative acute hyperglycemia, and the evidence for predicting adverse outcomes of stroke continues to be strengthened. Using the secondary analysis conducted in the Intensive Statin and Antiplatelet Therapy for High-risk Intracranial or Extracranial Atherosclerosis (INSPIRES) randomized trial as an example (24), an elevated SHR was independently correlated with the risk of multiple adverse outcomes. Therefore, the observed negative association between HGI and LAA should not be interpreted purely as evidence of a stable glycation phenotype. Because HGI was calculated using admission FPG, acute stress-related elevation of FPG may reduce the calculated HGI when HbA1c does not increase synchronously. In this sense, low HGI may overlap conceptually with relative stress hyperglycemia. However, HGI and the stress hyperglycemia ratio (SHR) are not identical indicators. SHR directly expresses admission glucose relative to HbA1c-estimated chronic glycemia, whereas HGI represents the residual difference between measured HbA1c and FPG-predicted HbA1c. Both indicators are derived from FPG and HbA1c and may therefore capture related but not completely equivalent aspects of FPG–HbA1c discordance. In the present study, we interpreted HGI primarily as a risk-stratification marker in the acute stroke setting rather than as a purely independent biological glycation phenotype. This mechanistic explanation is also consistent with the finding in this study that a low HGI indicates a high risk of in-hospital mortality. From the perspective of pathophysiology, the occurrence and development of LAA are closely related to the severe burden of atherosclerosis, systemic inflammatory responses, vascular endothelial dysfunction and ischemia–reperfusion injury; these pathological processes can overactivate neuroendocrine–inflammatory stress pathways, thereby triggering more pronounced hyperglycemia during the acute phase (25, 26). The additional SHR sensitivity analysis further supports this interpretation. HGI and SHR were strongly inversely correlated in both cohorts, and higher SHR showed directionally consistent associations with increased LAA risk and in-hospital mortality. These findings suggest that the low-HGI signal in the acute stroke setting is unlikely to represent a purely stable glycation phenotype. Rather, it may substantially reflect acute FPG–HbA1c discordance related to stress hyperglycemia. Therefore, HGI should be interpreted as a pragmatic risk-stratification marker rather than as a distinct biological mechanism independent of stress hyperglycemia.

In addition to acute fluctuations in the glucose levels, the HbA1c level is affected by a variety of nonglycemic factors (such as anemia, abnormal renal function, inflammation, hemoglobin level variation, and erythrocyte lifespan/turnover), and these factors can cause HbA1c levels to deviate from the true average blood glucose level, thereby altering the numerical value and biological significance of the HGI (27). In recent years, studies (28) have attempted to correct the HbA1c level using the erythrocyte lifespan, which suggests that variations in erythrocyte lifespan among individuals may lead to systematic bias in the HbA1c level when reflecting intracellular glucose exposure levels. This finding indicates that in the acute phase of stroke, if patients with different etiological subtypes have differences in anemia, renal function, or inflammatory load, these factors may change the correlation between HbA1c levels and blood glucose levels, thereby affecting the strength of association between the HGI and LAA. Although the key clinical confounding factors were adjusted in the multivariate model in this study, future studies still need to perform more systematic control or stratified analyses on these determinants at the design stage. Mechanistically, these potential influencing factors can be divided into two categories: Glucose metabolism-related pathways and erythrocyte biology-related pathways. Erythrocyte-related genetic variations can directly affect HbA1c levels without affecting average blood glucose levels, thereby affecting derived indicators, including the HGI (29). This mechanism is highly important for explaining the MR results of this study. If the genetic instrument variables used for calculation of the HGI contain a significant number of SNPs in genes belonging to the erythrocyte pathway, this may not only introduce horizontal pleiotropy bias associated with atherosclerosis or stroke but also weaken the instrument variables’ representativeness of the core causal chain linking glucose metabolism to atherosclerosis. Ultimately, this could result in non-significant conclusions from the MR analyses.

From the perspective of causal inference, the MR findings should be interpreted cautiously. Although several MR estimators showed directionally negative point estimates, the primary IVW result was not statistically significant, and the 95% confidence interval crossed the null value. Therefore, the present MR analysis does not establish a strong causal genetic relationship between HGI and LAA risk. In addition, the HGI GWAS used for instrument selection had limited sample size, and HGI-related genetic variants may partly reflect erythrocyte biology or hemoglobin metabolism rather than glucose metabolism alone. Accordingly, the MR findings should be regarded as exploratory and hypothesis-generating.

## 5 Study limitations

This study has several limitations. First, owing to its retrospective observational design, causal inference cannot be established, and residual confounding remains possible, particularly because stroke severity and critical illness severity indicators such as NIHSS, Glasgow Coma Scale, SOFA score, mechanical ventilation, and vasopressor use were not consistently available. Second, HGI was calculated using the first post-admission FPG and HbA1c values; therefore, low HGI may partly reflect acute stress hyperglycemia, treatment-related glucose changes, or FPG–HbA1c discordance rather than a purely stable glycation phenotype. Although the additional SHR sensitivity analysis supported substantial overlap between low HGI and stress-hyperglycemia-related FPG–HbA1c discordance, the retrospective design did not allow us to fully separate chronic glycation phenotype from acute stress hyperglycemia. Third, the MIMIC-IV cohort was identified using ICD-coded ischemic stroke diagnoses, and stroke was not necessarily the primary reason for admission, which may have introduced clinical heterogeneity; moreover, the external validation cohort was single-center and relatively small. Fourth, the HGI equations were cohort-specific, several lipid-related variables had moderate missingness, and complete-case analysis may have introduced selection bias. Finally, the MR analysis was statistically non-significant and should be interpreted as exploratory, and the mortality endpoint was all-cause in-hospital mortality rather than stroke-specific mortality.

## 6 Conclusion

In summary, this study found that low HGI was associated with higher LAA risk and increased in-hospital all-cause mortality in patients with IS, with nonlinear dose–response patterns. The additional SHR sensitivity analysis indicated that this low-HGI signal substantially overlaps with stress-hyperglycemia-related FPG–HbA1c discordance. Therefore, HGI may serve as an accessible risk-stratification marker in patients with IS, but it should not be interpreted as evidence of a distinct or causal glycation phenotype independent of stress hyperglycemia. The MR analysis did not reach statistical significance and therefore does not establish a strong causal genetic relationship between HGI and LAA; further confirmation in larger genetic studies and prospective stroke cohorts is warranted.

## Data Availability

The original contributions presented in the study are included in the article/Supplementary material, further inquiries can be directed to the corresponding author/s.
