# Supplementary material for: Association of the hemoglobin glycation index with the large-artery atherosclerosis subtype in ischemic stroke: a dual-cohort study
Source: Front Neurol. 2026 Jun 29;17:1841295. doi: 10.3389/fneur.2026.1841295 (PMC13357138; doi:10.3389/fneur.2026.1841295)
Supplement: Supplementary file 1 [file Table_1.DOCX]

**Table S1 International Classification of Diseases (ICD-9 and ICD-10) Coding Standards**

| **Database** | **Retrieval Strategy** |
| --- | --- |
| ICD-9 | 43301, 43311, 43321, 43331, 43381, 43391, 43401, 43411, 43491, 436 |
| ICD-10 | I630, I631, I632, I633, I634, I635, I636, I638, I639 |
