# Supplementary material for: Association of the hemoglobin glycation index with the large-artery atherosclerosis subtype in ischemic stroke: a dual-cohort study
Source: Front Neurol. 2026 Jun 29;17:1841295. doi: 10.3389/fneur.2026.1841295 (PMC13357138; doi:10.3389/fneur.2026.1841295)
Supplement: Supplementary file 2 [file Table_2.DOCX]

Supplementary Table S2. SHR sensitivity analyses and correlation with HGI

Part A. Spearman correlation between HGI and SHR

| **Cohort** | **Variables** | **Spearman’s ρ** | **P value** |
| --- | --- | --- | --- |
| MIMIC-IV | HGI vs SHR | −0.711 | <0.001 |
| Clinical validation cohort | HGI vs SHR | −0.723 | <0.001 |

Part B. Parallel fully adjusted models using HGI and SHR

| **Cohort** | **Outcome** | **Exposure** | **Model** | **Adjusted OR (95% CI)** | **P value** |
| --- | --- | --- | --- | --- | --- |
| MIMIC-IV | LAA | HGI | Model 3 | 0.579 (0.498–0.671) | <0.001 |
| MIMIC-IV | LAA | SHR | Model 3 | 6.287 (4.201–9.416) | <0.001 |
| Clinical validation cohort | LAA | HGI | Model 3 | 0.599 (0.465–0.755) | <0.001 |
| Clinical validation cohort | LAA | SHR | Model 3 | 13.957 (4.475–48.450) | <0.001 |
| MIMIC-IV | In-hospital all-cause mortality | HGI | Model 3 | 0.488 (0.379–0.628) | <0.001 |
| MIMIC-IV | In-hospital all-cause mortality | SHR | Model 3 | 11.799 (6.053–22.984) | <0.001 |

Abbreviations: CI, confidence interval; eAG, estimated average glucose; FPG, fasting plasma glucose; HGI, hemoglobin glycation index; LAA, large-artery atherosclerosis; SHR, stress hyperglycemia ratio. SHR was calculated as FPG/eAG, where eAG = 28.7 × HbA1c − 46.7. Model 3 adjusted for age, sex, hypertension, chronic kidney disease, type 2 diabetes mellitus, ischemic heart disease, atrial fibrillation, platelet count, hemoglobin, red blood cell count, white blood cell count, triglycerides, creatinine, blood urea nitrogen, total cholesterol, low-density lipoprotein cholesterol, and high-density lipoprotein cholesterol. ORs for continuous HGI and SHR are per 1-unit increment and are not directly comparable because the two variables have different numerical scales.
